# Retinal pericytes and cytomegalovirus infectivity: implications for HCMV-induced retinopathy and congenital ocular disease

**DOI:** 10.1186/s12974-014-0219-y

**Published:** 2015-01-09

**Authors:** Irene Wilkerson, Joshua Laban, Johnathan M Mitchell, Nader Sheibani, Donald J Alcendor

**Affiliations:** Department of Microbiology and Immunology, Center for AIDS Health Disparities Research, Meharry Medical College, School of Medicine, 1005 Dr DB Todd Jr Blvd, Nashville, TN 37208 USA; Department of Ophthalmology and Visual Sciences, University of Wisconsin School of Medicine and Public Health, Madison, WI 53792 USA

**Keywords:** Cytomegalovirus, Pericytes, Retina, Blood-brain barrier, Cytokines, Inflammation, Angiogenesis

## Abstract

**Background:**

Human cytomegalovirus (HCMV) is the leading infectious cause of vision loss among congenitally infected children. Retinal pericytes play an essential role in maintaining retinal vascular and endothelial cell proliferation. However, the role of retinal pericytes in ocular HCMV pathogenesis is unknown.

**Methods:**

Retinal pericytes were exposed to clinical (SBCMV) and lab strains of HCMV; infectivity was analyzed by microscopy, immunofluorescence and qRT-PCR (reverse transcription polymerase chain reaction). Cytokine expression was examined by Luminex assay. Recombinant HCMV-GPF was used to examine viral replication kinetics. A Tricell culture model of the inner blood-retinal barrier (IBRB) was examined for cell type infectivity using immunohistochemistry.

**Results:**

Retinal pericytes expressed the biomarker neuron-glial antigen 2. Antigenic expression profiles for several cytoskeletal, cell adhesion and inflammatory proteins were shared by both retinal and brain pericytes. Infected pericytes showed cytomegalic cytopathology and expressed mRNAs for the major immediate protein (MIE) and HCMV phosphorylated envelop protein 65. qRT-PCR analysis showed full lytic replication of HCMV in retinal pericytes. Pericytes exposed to SBCMV for 9 days expressed higher levels of vascular endothelial cell growth factor mRNA compared to controls. Luminex analysis of supernatants from SBCMV-infected retinal pericytes had increased levels of macrophage inflammatory protein-1α, beta-2 microglobulin (B2-m), matrix metalloproteinase-3 and -9 (MMP3/9), and lower levels of IL-6 and IL-8 compared to controls. At 24 hours post infection, pericytes expressed higher levels of IL-8, TIMP-1 (tissue inhibitor of metalloproteinase-1), and RANTES (regulated upon activation normal T cell-expressed and presumably secreted) but lower levels of MMP9. Time course analysis showed that both brain and retinal pericytes were more permissive for HCMV infection than other cellular components of the BBB (blood-brain barrier) and IBRB. Using a Tricell culture model of the IBRB (retinal endothelial, pericytes, Müller cells), retinal pericytes were most permissive for SBCMV infection. SBCMV infection of this IBRB Tricell mixture for 96 hours resulted in increased levels of IL-6, MMP9, and stem cell factor with a concomitant decrease in granulocyte-macrophage colony-stimulating factor and TNF-alpha.

**Conclusion:**

In retinal pericytes, HCMV induces proinflammatory and angiogenic cytokines. In the IBRB, pericytes likely serve as an amplification reservoir which contributes to retinal inflammation and angiogenesis.

## Background

Human cytomegalovirus (HCMV) is an opportunistic pathogen that is known to cause life-threatening disease in immunocompromised individuals such as neonates, transplant patients and sufferers of HIV/AIDS [1]. Congenital HCMV infection is the major cause of birth defects, affecting approximately 40,000 children (0.2 to 2% of all live births) in the United States each year [2-6], and is the leading infectious cause of mental retardation and deafness in children [7,8]. Central nervous system (CNS) abnormalities in newborn babies can include vision loss, mental retardation, motor deficits, seizures and sensorineural hearing loss [9-11]. With only 10 to 15% of children presenting with symptomatic disease at birth, HCMV can cause long-term progressive neuropathology in children who are asymptomatic at birth. It is estimated that approximately 8,000 children are affected each year with some form of neuropathology associated with congenital HCMV infections in the United States [12]. Ophthalmic presentations associated with HCMV-induced retinitis have been reported [13,14]; however, HCMV dissemination in the inner blood-retinal barrier (IBRB) remains unclear. Retinitis due to HCMV infection, which can also result in blindness, is the most prevalent ocular disease in individuals with HIV/AIDS [15-18].

The retina and brain have the highest density of vascular pericytes in the body [19]. Alcendor *et al.* recently reported that primary human brain vascular pericytes were fully permissive for HCMV infection, were more permissive for HCMV lytic replication compared to brain microvascular endothelial cells (BMVEC) or astrocytes, and could serve as amplification reservoirs for HCMV infection and dissemination in the CNS [20]. In addition, pericyte exposure to HCMV induced a proinflammatory cascade that likely contributes to neuroinflammation [20]. The eye is the outermost extension of the CNS, and the IBRB [21,22] shares topological similarities to the blood-brain barrier (BBB), namely that the neurovascular unit includes retinal pericytes, retinal microvascular endothelial cells and Müller cells. Retinal pericytes play an essential role in maintaining retinal vascular and endothelial cell proliferation [23]. The role of retinal pericytes in HCMV-induced ocular disease is currently unknown. It is important to identify the role of retinal pericytes and their contribution to HCMV infection and dissemination. To our knowledge, this is the first report to date that investigates the infectivity of human retinal pericytes for HCMV and their potential role in viral dissemination in the IBRB and the concomitant implications for HCMV-associated ocular disease. Our hypothesis is that in vascular beds normally trafficked by HCMV during primary infection that includes the brain and retinal barriers, pericytes are the most permissive cell type within these vascular beds for HCMV infection and represent the cell type responsible for virus amplification and dissemination and greatly contribute to altering these microenvironments via the induction of proinflammatory and angiogenic cytokines.

## Methods

### Cells and viruses

The SBCMV clinical strain was obtained from Dr. Ravit Boger (Johns Hopkins University) [20] and the HCMV-GFP recombinant virus was obtained from Dr. Gary Hayward (Johns Hopkins University). Acquisition of the “SBCMV” clinical isolate of was approved by the Internal Review Board and Ethics Committee of Johns Hopkins University Medical Center in Baltimore, Maryland. Primary human retinal capillary endothelial cells, retinal pericytes, human brain microvascular endothelial cells, human brain pericytes and human astrocytes were obtained from Cell Systems Corporation (Kirkland, WA, USA) and were cultivated in Pericyte Media (PM) from ScienCell (Carlsbad, CA, USA). The human Müller cell line MIO-M1 [24], derived from an adult retina, was kindly provided by Dr. John Penn (Vanderbilt University Medical Center Eye Institute). Acquisition of the MIO-M1 cell line was approved by the Internal Review Board and Ethics Committee of Vanderbilt University Medical Center in Nashville, Tennessee. The Retinal pericytes were maintained at low passage in PM media. Cells were trypsinized and plated in uncoated 100- cm^2^ dishes or uncoated 4.2-cm^2^/well glass chamber slides at a density of 1 × 10^6^ and 2.5 × 10^5^ cells per dish and well, respectively. Heat-killed SBCMV was prepared by heating the viral inoculum to 65°C for 30 minutes in a water bath [25]. Heat-killed virus was used as a replication control in place of UV inactivated virus. The heating protocol for HCMV that we use is mild and is unlikely to completely destroy the viral envelope.

### Cytomegalovirus infection of retinal pericytes and RNA isolation

Cytomegalovirus infection and RNA isolation procedures have been previously described [20]. The SBCMV clinical isolate, HCMV Towne strain, and the HCMV-GFP recombinant virus were all cultivated in human foreskin fibroblast (HFF) cells. Retinal pericytes were infected at a multiplicity (moi) of 0.1, virus adsorption was allowed for 2 to 3 hours and the infectious inoculum was removed and replaced with fresh media.

### Immunofluorescence

Chamber slide cultures containing either mock infected or infected cells were washed twice with PBS, pH 7.4, air dried, and fixed in absolute methanol for 10 minutes. Cells were air dried for 15 minutes, hydrated in Tris saline (pH 7.4) for 5 minutes, and then incubated for 1 hour with monoclonal antibodies diluted 1:50 in PBS, pH 7.4. Antibodies to monitor tissue markers in uninfected human retinal and brain vascular pericytes included the following from Santa Cruz Biotechnology (Santa Cruz, CA, USA): fibronectin, vimentin, CD68, NG2 proteoglycan, beta catenin, smooth muscle actin, vascular cell adhesion molecule-1 (VCAM-1), vascular endothelial cadherin (VE-cadherin), alpha 4 integrin, TNF-alpha, and NFkB. Those antibodies obtained from Millipore (Temecula, CA, USA) included: platelet endothelial cell adhesion molecule-1 (PECAM-1), intercellular adhesion molecule-1 (ICAM-1), melanoma cell adhesion molecule-1 (MelCAM-1), endothelial-leukocyte adhesion molecule 1 (E-selectin), von Willebrand factor (VWF), and Tissue Factor. Finally, RANTES was obtained from R&D Systems (Minneapolis, MN, USA). All antibodies were diluted 1:50 in PBS, pH 7.4.

For HCMV infections, retinal pericytes were incubated for 1 hour with monoclonal antibodies to HCMV major immediate protein (MIE) at a 1:50 dilution (MIE, mAb810, Millipore. Temecula, CA, USA), the HCMV tegument protein pp65 (UL83) at 1:50 (Vector Laboratories, Burlingame, CA, USA) or the HCMV late nuclear antigen pp28 (Santa Cruz Biotechnology, Santa Cruz, CA, USA). Cells were washed 3 times with Tris saline and then incubated at 37°C for 30 minutes with a combination of secondary donkey anti-mouse IgG antibodies conjugated with fluroescein isothiocyanate (FITC, Jackson ImmunoResearch, West Grove, PA, USA) at a 1:100 dilution in PBS. Cells were washed another 3 times in Tris saline and mounted with Vectashield mounting media (Burlingame, CA, USA) containing 1.5 μg/ml of 4′,6-diamidino-2-phenylindole (DAPI). Fluorescence was photographed with a Nikon TE 2000S fluorescent microscope (Melville, NY, USA) mounted with a charge-coupled device (CCD) camera.

### Real-time qPCR

Total RNA was extracted from SBCMV-infected retinal pericytes, mock infected and heat-killed SBCMV (control) using a Qiagen RNAeasy Mini Kit (Qiagen, Valencia, CA, USA). RNA was then DNAase treated before elution from the column according to the manufacturer’s recommendations. Messenger RNA in 0.5 μg of each sample was primed using oligo-dT and reverse transcribed with a high capacity cDNA reverse transcription kit (Applied Biosystems, Foster City, CA, USA). Real-time PCR was performed as previously described [20] by using gene-specific primers for HCMV MIE: forward 5′CCAAGCGGGCTCTGATAACCAAGCC3′ and reverse 5′ CAGCACCATCCTCCTCTTCCTCTGG3′) and HCMV pp65: forward 5′GACACAACACCGTAAAGC3′ and reverse 5′CAGCGTTCGTGTTTCC3′). For amplification of vascular endothelial growth factor (VEGF^165^) we used the following primer pairs: forward 5′ATCTTCAAGCCATCCTGTGTCC3′ and reverse 5′CAAGGCCCACAGGGATTTTC3.

### IBRB Tricell culture infection model

A Tricell culture infection model of the IBRB, composed of primary human retinal microvascular endothelial cells, retinal pericytes and Müller cells, was established in chamber slides at a ratio of 3:1:1, respectively. The starting cell population ratios change during growth in culture; therefore, we consistently use these primary cells at the same passage level and the initial cultivations are performed with media recommended by the manufacturer. Retinal microvascular endothelial cells were initially cultivated in complete EBM-2 media (Lonza, Walkersville, MD, USA) and allowed to become confluent at a cell density 2.5 x 10^5^. Retinal pericytes were then added and the dual mixture was then cultivated in PM. After 48 hours, Müller cells were added to complete the Tricell mixture growing in PM. The Tricell mixture was then infected for 96 hours with the SBCMV clinical isolate at a moi of 0.1. Cell supernatants were analyzed by Luminex assay [26]. The Tricell mixture was then stained for viability using a live/dead cell viability assay kit (Life Technologies, Grand Island, NY, USA).

### Immunohistochemistry

Dual labeled immunohistochemistry (IHC) was performed as previously described [27].

A monoclonal antibody to the human retinal endothelial cell antigenic biomarker VWF was used to stain retinal endothelial cells. Endothelial cells were visualized in the mixture using Vector VIP (Vector Laboratories, Burlingame, CA, USA) as a peroxidase substrate. Antigen blocking between the use of different substrates was performed using an Avidin/Biotin blocking kit (Vector Laboratories, Burlingame, CA, USA). A rabbit polyclonal antibody to the pericyte antigenic biomarker NG2 proteogylcan (Abcam, Cambridge, MA, USA) was used to stain retinal pericytes. Retinal pericytes were visualized in the Tricell mixture using Vector SG (Vector Laboratories, Burlingame, CA, USA). A rabbit polyclonal antibody to the Müller cell antigenic biomarker glial fibrillary acidic protein (GFAP, Abcam, Cambridge, MA, USA) was used to stain Müller cells. Müller cells were visualized in the mixture using diaminobenzidine (DAB) as a peroxidase substrate (Vector Laboratories, Burlingame, MA, USA). All labeling and substrate preparations were performed in accordance with the manufacturers’ recommendations.

### Luminex analysis

The inflammatory cytokine analysis was performed with 200 μl of cell supernatants from mock infected, SBCMV-infected and SBCMV (heat-killed) retinal pericytes 9 days post exposure using a Luminex instrument (Luminex Corporation, Austin, TX, USA) and a 100- plate viewer software. The inflammatory cytokine panel (Inflammation MAP v1.0.) was designed to measure 47 proinflammatory and angiogenic cytokines. All specimens, standards and controls were run in triplicate according to the manufacturer’s protocol. The instrument is configured to collect a minimum of 100 beads per region [28].

### Statistical analysis

Experiments presented in this study were performed in triplicate. Mock infected, SBCMV-infected and heat-killed SBCMV-exposed retinal pericyte cell pellets obtained 9 days post infection were used for qRT-PCR. qRT-PCR experiments were replicated three times and normalized to glyceraldehyde 3-phosphate dehydrogenase (GAPDH). A *P-*value of < 0.05 was considered statistically significant.

## Results

### Expression profiles of human retinal and brain pericytes are shared

**Figure 1:**
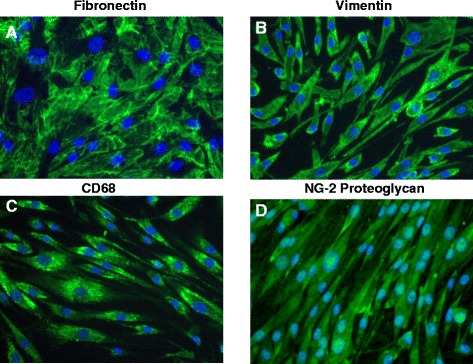
**Normal retinal pericyte expression markers.** Immunofluorescent stained images of retinal pericytes of: **(A)** a confluent monolayer of retinal pericytes staining positive for fibronectin, and retinal pericytes staining positive for **(B)** vimentin, **(C)** CD68 and **(D)** NG2 proteogylcan. All images were taken on a Nikon TE2000S microscope mounted with a charge-coupled device (CCD) camera at a 200x magnification. 4',6-diamidino-2-phenylindole (DAPI) was used to stain the nuclei blue.

Immunoflorescent staining confirms that normal primary human retinal pericytes express pericyte biomarkers (Figure 1). Retinal pericytes were shown to stain positive for fibronectin (Figure 1A), vimentin (Figure 1B), CD68 (Figure 1C) and NG2 proteogylcan (Figure 1D). This expression pattern was consistent with the profile we observed with primary human brain pericytes [20]. We then compared the expression profiles of normal primary human retinal pericytes versus brain pericytes by immunofluorescent staining for specific cytoskeletal, cellular adhesion and inflammatory biomarkers (Table 1). Pericytes were examined for expression of cytosketal antigenic biomarkers (fibronectin, vimentin, beta catenin, smooth muscle actin, NG2 proteoglycan), cellular adhesion antigenic biomarkers (CD68, PECAM-1, VCAM-1, ICAM-1, MelCAM-1, E-selectin, VWF, alpha 4 integrin) and inflammatory biomarkers (TNF-alpha, NFkB, RANTES and Tissue Factor). We found a high degree of similarity in the antigenic expression profiles in human brain and retinal pericytes for those cytoskeletal, cell adhesion and inflammatory biomarkers we tested (Table 1). However, we observed a lower expression level of alpha smooth muscle actin and a higher level of VCAM-1 in retinal pericytes compared to brain pericytes. The staining profile of human retinal pericytes for key antigenic biomarkers (fibronectin, vimentin, CD68, and NG2 proteoglycan) was similar to levels previously shown to be expressed in human brain pericytes [20].

**Table 1:** **Antigenic biomarkers expressed by human brain and retinal pericytes**

		**Brain pericytes**	**Retina pericytes**
	**Fibronectin**	**+**	**+**
**Cytoskeletal**	**Vimentin**	**+**	**+**
	**Beta catenin**	**−**	**−**
	**Smooth muscle actin**	**+**	**+/−**
	**NG2 proteoglycan**	**+**	**+**
	**CD68**	**+**	**+**
	**PECAM-1**	**−**	**−**
	**VCAM-1**	**+/−**	**−**
**Cellular adhesion**	**ICAM-1**	**−**	**−**
	**MelCAM-1**	**−**	**−**
	**E-selectin**	**−**	**−**
	**VE cadherin**	**−**	**−**
	**von Willebrand factor**	**−**	**−**
	**Alpha 4 integrin**	**−**	**−**
	**TNF-alpha**	**−**	**−**
**Inflammation**	**NFkB**	**−**	**−**
	**RANTES**	**−**	**−**
	**Tissue Factor**	**−**	**−**

Dashes indicate a negative result for immunofluorescence staining.

### Retinal pericytes are fully permissive for HCMV infection

**Figure 2:**
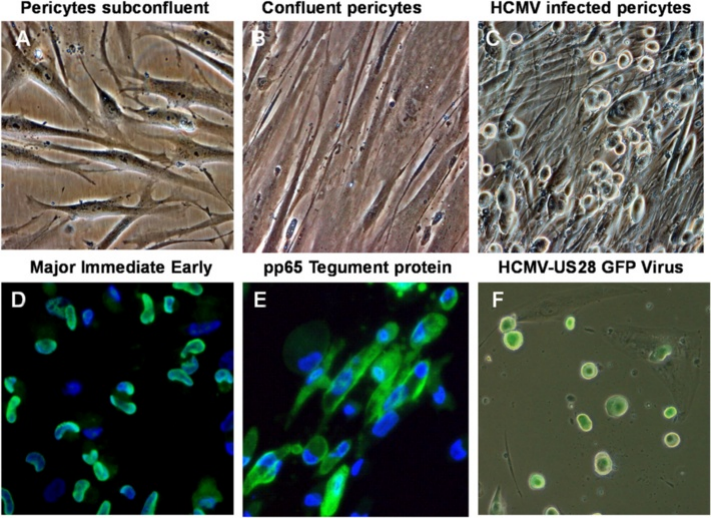
**Human cytomegalovirus (HCMV) infectivity of primary human retinal pericytes.** Phase contrast images of **(A)** an uninfected subconfluent monolayer of retinal pericytes, **(B)** a confluent monolayer of retinal pericytes and **(C)** pericytes 10 days after infection with the clinical strain SBCMV. Immunofluorescence staining of SBCMV-infected retinal pericytes for **(D)** major immediate protein (MIE) protein and **(E)** pp65 late stage protein. **(F)** Phase fluorescent overlay image of human retinal pericytes infected with a recombinant HCMV virus expressing GFP. All images were taken on a Nikon TE2000S microscope (200x magnification).

**Figure 3:**
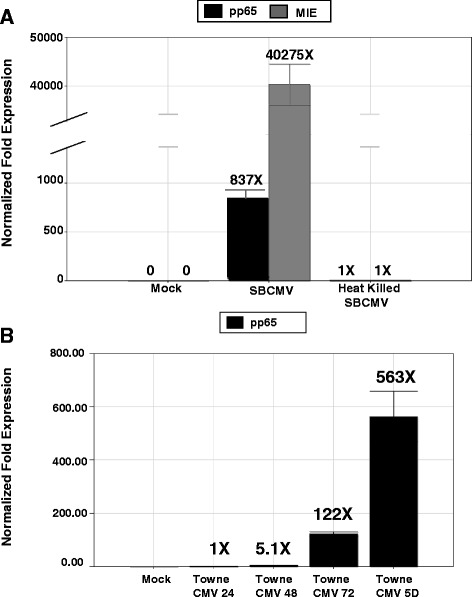
**Human cytmegalovirus (HCMV) replication kinetics in human retinal pericytes. (A)** Expression of HCMV mRNA by qRT-PCR in human retinal pericytes exposed to SBCMV-infected and heat-killed virus compared to mock infected controls. Human retinal pericytes were exposed to a human primary clinical isolate of HCMV, designated ‘SBCMV’, for 10 days. Total RNA was extracted from infected cells followed by cDNA amplification and qRT-PCR. **(B)** qRT-PCR was performed using mRNA from human retinal pericytes exposed to the Towne strain of HCMV after 24, 48, 72 hours and 5 days postinfection for the HCMV pp65 late protein mRNA transcripts. Fold expression was normalized to glyceraldehyde 3-phosphate dehydrogenase (GAPDH). Error bars represent the standard error of the mean (SEM) on triplicate experiments.

Primary retinal pericytes showed similar morphological characteristics to those shown by brain vascular pericytes, namely a long extension of the cytoplasm that was clearly visible in sub-confluent cultures (Figure 2A) [20]. However, when confluent they appeared fibroblastic in appearance (Figure 2B). Using a low moi with the SBCMV clinical isolate, we observed characteristic HCMV cytomegalic cytopathology 10 days post infection (Figure 2C). We also demonstrated that retinal pericytes were fully lytic for HCMV replication by expression of HCMV MIE 1 and 2 and the late viral tegument protein pp65/UL83 (Figures 2D and E). Infection of retinal pericytes with an HCMV-US28 recombinant virus expressing GFP showed that retinal pericytes support HCMV lytic replication with more than 90% of cells with cytomegalic cytopathology 6 days post infection (Figure 2F). We also examined viral replication kinetics by monitoring the expression of HCMV mRNA transcription by qRT-PCR in human retinal pericytes exposed to SBCMV-infected (clinical strain) and heat-killed virus (Figure 3A). Ten days post infection using the SBCMV clinical isolate we observed a > 40,000 fold and 837-fold increase in HCMV MIE and pp65 mRNA, respectively, compared to mock infected and heat-killed virus controls (Figure 3A). Using the Towne strain of HCMV we examined the temporal expression of the pp65 virion tegument protein mRNA in infected retinal pericytes. We observed a 1-fold, a 5.1-fold, a 122-fold and a 563-fold increase in pp65 mRNA post infection in retinal pericytes at 24, 48, 72 hours and 5 days, respectively (Figure 3B). The highest level of HCMV transcription was observed in SBCMV-infected cells following transcriptional amplification of the major immediate genes MIE (IE1, IE2) [29] and the viral pp65 late tegument protein [30] (Figure 3A). Higher levels of MIE transcription were observed compared to pp65 expression levels. No significant virus transcripts were observed in mock or heat-killed-exposed retinal pericytes (Figure 3A). Time course analysis of HCMV infection using the Towne strain of HCMV revealed a time-dependent increase in pp65 transcription consistent with permissive replication for cytomegalovirus (Figure 3B).

### Cellular dysregulation of angiogenic and proinflammatory cytokines in SBCMV-infected retinal pericytes

**Figure 4:**
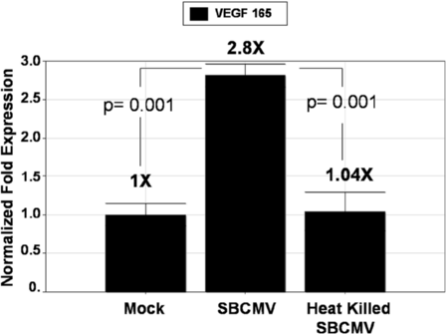
**SBCMV induction of vascular endothelial cell growth factor (VEGF) in human retinal pericytes.** Expression of VEGF^165^ mRNA by qRT-PCR in human retinal pericytes exposed to SBCMVinfected and heat-killed virus. Human pericytes were exposed to a human primary clinical isolate of HCMV, designated ‘SBCMV’ for 5 days. Total RNA was extracted from infected cells, followed by cDNA amplification and qRT-PCR. Fold expression was normalized to glyceraldehyde 3-dehydrogenase (GAPDH). Experiments were performed in triplicate and error bars represent the standard error of the mean (SEM). A *P* < 0.05 was considered significant.

**Figure 5:**
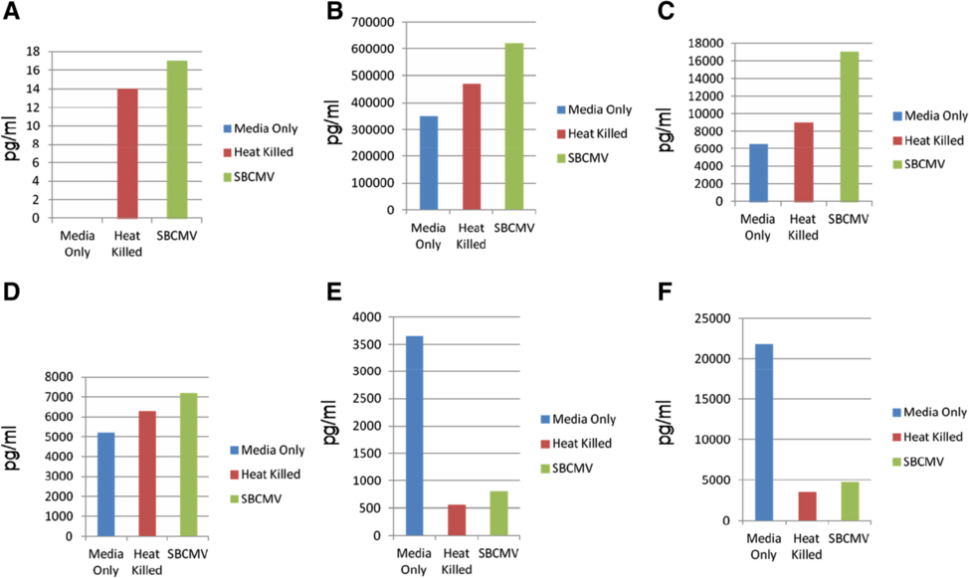
**SBCMV induction of proinflammatory and angiogenic cytokines at 9 days.** Cytokine profiles of SBCMV-infected pericytes by Luminex analysis at 9 days post infection are given. Results from cells exposed to media only are shown as blue bars, cells exposed to heat-killed SBCMV are shown as red bars and results from cells exposed to the SBCMV clinical isolate are shown as green bars. Results are included for **(A)** MIP-1α , **(B)** B2-m, **(C)** MMP3, **(D)** MMP9, **(E)** IL-6 and **(F)** IL-8. Results are given in pg/ml. Results shown are the averages of triplicate samples.

The majority of retinal vasculopathies are associated with dysregulation of angiogenesis and inflammation [28]. We examined SBCMV-infected human retinal pericytes along with mock infected cells and cells exposed to heat-killed virus for changes in vascular endothelial cell growth factor (VEGF) expression (Figure 4). We observe a 2.8-fold increase in VEGF^165^ mRNA expression by qRT-PCR in SBCMV-infected cells when compared to mock infected and heat-killed virus-exposed human retinal pericytes. We also observed a marginal but insignificant increase in VEGF^165^ in pericytes exposed to heat-killed virus (Figure 4). Transcription analysis using VEGF primers that recognized all of the major three splice variants were observed to be upregulated in SBCMV-infected pericytes compared to uninfected control cells (data not shown). We then made a comparative analysis using Luminex assays of 9-day supernatants from the above mentioned cells that exhibited evidence of cytopathology (Figure 5). We observed a high level of MIP-1α secretion by retinal pericytes exposed to both SBCMV and to heat-killed virus compared to mock infected cells (Figure 5A). The highest level of MIP-1α secretion was observed in pericytes exposed to replication competent virus. High levels of B2-m were observed in supernatants from pericytes exposed to SBCMV and heat-killed virus compared to uninfected cells (Figure 5B), although the highest level of B2-m was observed in SBCMV-infected pericytes. Increased levels of MMP3 and MMP9 were observed in both 9-day SBCMV-infected and SBCMV heat-killed-exposed pericytes compared to uninfected controls (Figure 5C and D), although levels in SBCMV-treated cells were higher than cells exposed to heat-killed virus. However, we observed reduced levels of the proinflammatory cytokines IL-6 and IL-8 in both SBCMV-infected and SBCMV heat-killed-exposed pericytes compared to uninfected controls (Figure 5E and F), with the greatest reduction occurring in heat-killed virus exposed cultures.

**Figure 6:**
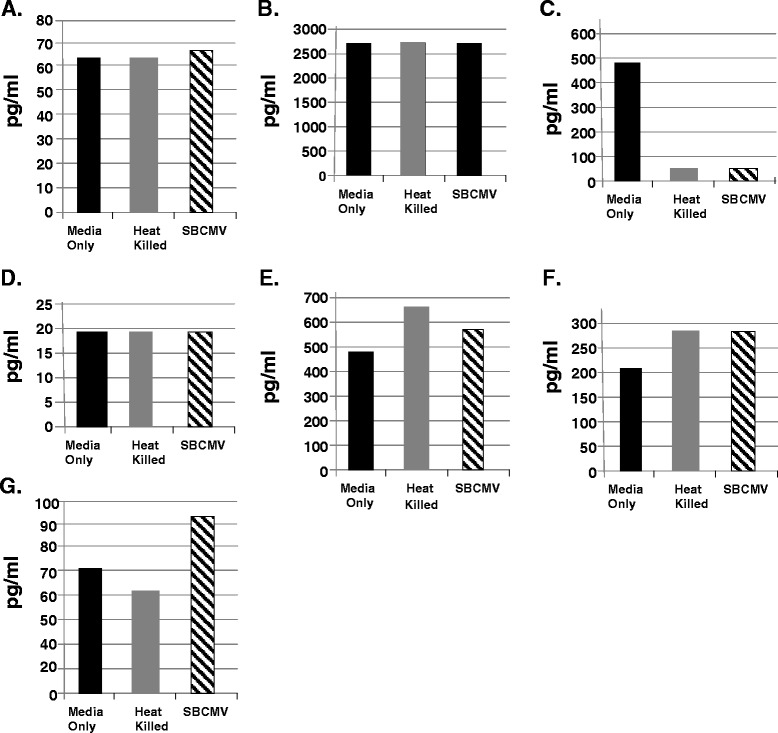
**SBCMV induction of proinflammatory and angiogenic cytokines at 24 hours.** Cytokine profiles of SBCMV-infected pericytes (only) by Luminex analysis at 24 hours post infection are given. Results from cells exposed to media only are shown as solid black bars, cells exposed to heat-killed SBCMV are shown as gray bars and results from cells exposed to the SBCMV clinical isolate are shown as stippled black bars. Results are included for **(A)** B2-m, **(B)** MMP3, **(C)** MMP9, **(D)** IL-6 **(E)** IL-8, **(F)** TIMP-1, and **(G)** RANTES. Results are given in pg/ml. Results shown are the averages of triplicate samples.

At an earlier time point (24 hours post infection) we found no significant change in B2-m, MMP3, or IL-6 levels (Figure 6A, B, and D). We observed a decreased level of MMP9 in SBCMV-infected and heat-killed virus-exposed retinal pericytes compared to mock infected controls (Figure 6C). There was increase in IL-8, tissue inhibitor of mealloproteinase-1 (TIMP-1), and RANTES in SBCMV-infected retinal pericytes compared to mock infected controls (Figure 6E, F, and G). We observed a greater increase in IL-8 in heat-killed virus-exposed retinal pericytes compared to SBCMV-infected pericytes but a greater increase in RANTES in supernatants from SBCMV-infected pericytes compared to heat-killed virus-exposed retinal pericytes (Figure 6E and G). MIP-1α levels were undetectable at 24 hours in supernatants from SBCMV-infected and heat-killed virus-exposed retinal pericytes.

### Pericytes from human brain and retina are more permissive for HCMV than other cellular components of the BBB and the IBRB

**Figure 7:**
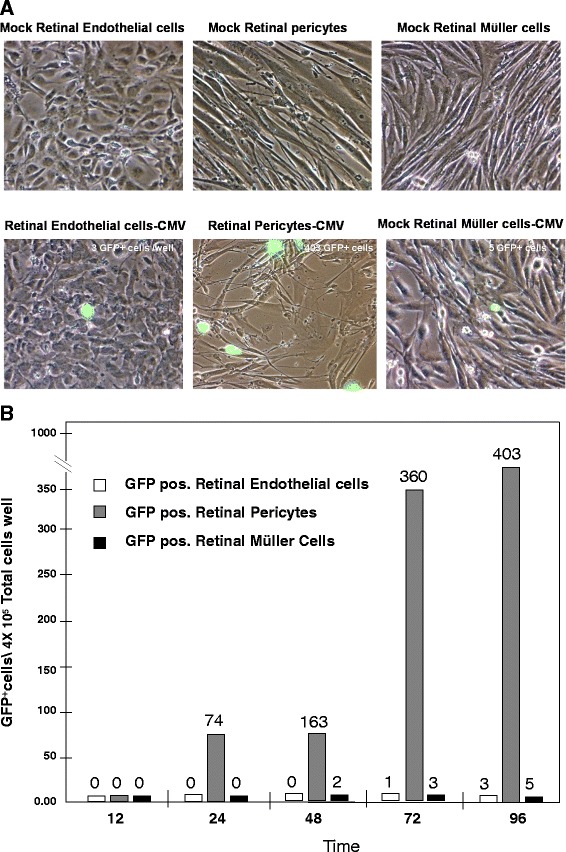
**Time course analysis of human cytomegalovirus-GFP (HCMV-GFP) infection of IBRB (inner blood-retinal barrier) cells. (A)** Top panel: phase contrast images of human mock and infected retinal microvascular endothelial cells, retinal pericytes and Müller cells. Bottom panel: phase contrast images of infected retinal microvascular endothelial cells, retinal pericytes and Müller cells with a fluorescent overlay showing HCMV-GFP-positive cells. Magnification = 200x. **(B)** A graph showing the number of infected HCMV-GFP-positive retinal microvascular endothelial cells (open bars), retinal pericytes (gray bars) and Müller cells (black bars) per 4 × 10^6^ total cells over the time course of 12, 24, 48 and 96 hours post infection.

**Figure 8:**
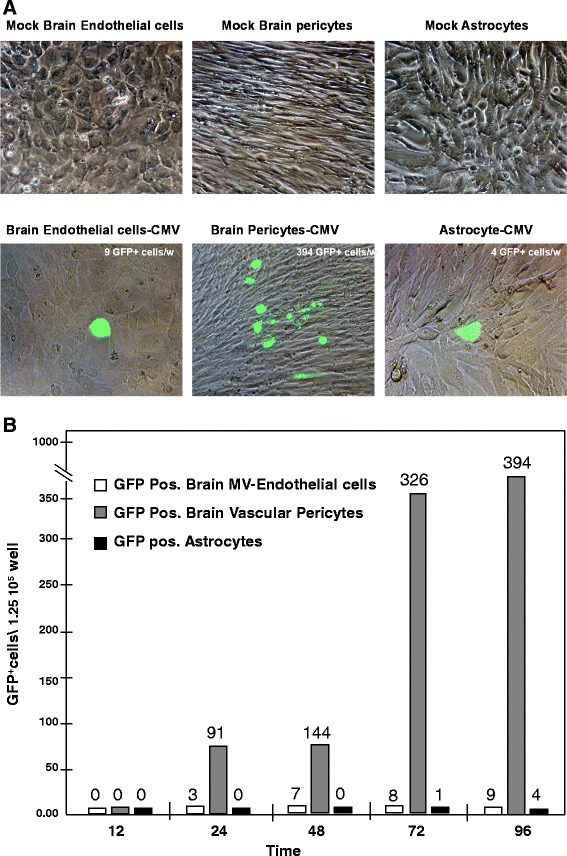
**Time course analysis of human cytomegalovius-GFP (HCMV-GFP) infection of BBB (blood-brain barrier) cells.** For comparison purposes, **(A)** the top panel includes phase contrast images of human mock and infected brain microvascular endothelial cells, brain vascular pericytes and astrocytes. The bottom panel shows phase contrast images of infected brain microvascular endothelial cells, brain pericytes and astrocytes with a fluorescent overlay showing HCMV-GFP-positive cells. Total magnification is 200x. **(B)** A graph indicating the number of infected HCMV-GFP-positive brain microvascular endothelial cells (open bars), brain pericytes (gray bars) and astrocytes (black bars) per 1.25 × 10^6^ total cells over the time course 12, 24, 48 and 96 hours post infection.

Cellular components of the human IBRB (includes retinal microvascular endothelial cells, retinal pericytes and Müller cells) as well as the cellular components of the human BBB (includes brain microvascular endothelial cells, vascular pericytes and astrocytes) were compared to determine their infectivity when exposed to a recombinant HCMV isolate encoding GFP. Individual cell types of both the IBRB and the BBB were infected with a moi of 0.1 to model *in vivo* clinical conditions; uninfected cells served as controls. Infections were performed in triplicate in chamber slides for 12, 24, 48 and 96 hours post infection (Figure 7A). The average total number of GFP-positive cells was counted by fluorescence microscopy. No GFP-positive cells were observed after 12 hours for the individual IBRB cell types but after 24 hours, pericyte cultures had a 74-fold, 160-fold, more than 300-fold and nearly a 400-fold increase in GFP-positive cells compared to Müller cells and retinal endothelial cells (Figure 7B). We observed no significant difference in this pattern of infectivity for the IBRB cell types at 10 days post infection (data not shown). Similar results were observed with cellular components of BBB showing brain pericytes as being more permissive for HCMV infection when compared to human brain microvascular endothelial cells or astrocytes (Figure 8A and B). No GFP-positive cells were observed after 12 hours for BBB cells, but at 24, 48, 72 and 96 hours, pericyte cultures had a roughly 90-fold, 140-fold, 325-fold and nearly 400-fold increase in GFP-positive cells, respectively, compared to brain microvascular endothelial cells or astrocytes (Figure 8A and B). Brain microvascular endothelial cells were consistently more permissive for HCMV than astrocytes or endothelial cells in this assay. We observed no significant difference in this pattern of infectivity for the IBRB cell types at 10 days post infection (data not shown).

### In a Tricell culture infection model, retinal pericytes are more permissive for SBCMV infection than retinal microvascular endothelial cells and Müller cells

**Figure 9:**
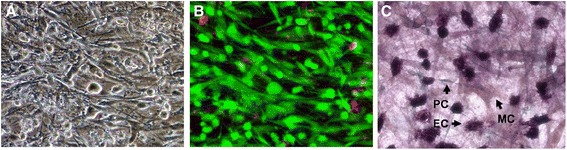
**A retinal Tricell culture model. (A)** Phase contrast image of a Tricell culture mixture of human retinal microvascular endothelial cells, pericytes and Müller cells representing the inner blood-retinal barrier (IBRB). **(B)** A live/dead stain of the retinal Tricell culture. **(C)** Triple labeled immunohistochemistry (IHC) of the IBRB cell culture (arrows indicate an endothelial (EC), pericyte (PC) and Müller cell (MC)). Magnifications = 200x.

**Figure 10:**
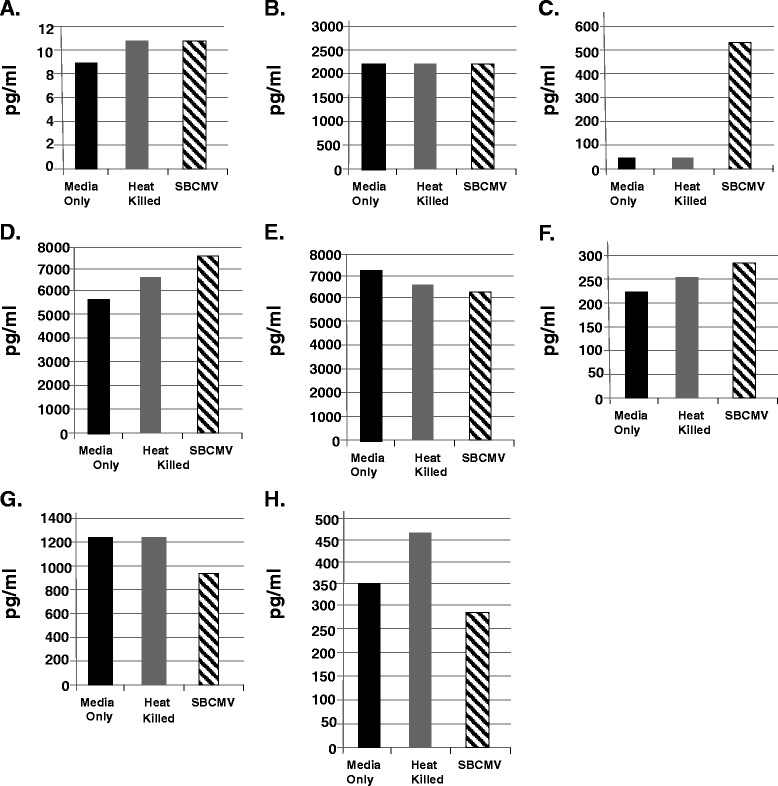
**SBCMV induction of proinflammatory and angiogenic cytokines in the inner blood-retinal barrier (IBRB) Tricell culture.** Cytokine profiles of SBCMV-infected pericytes by Luminex analysis at 96 hours post infection are given. Results from cells exposed to media only are shown as solid black bars, cells exposed to heat-killed SBCMV are shown as gray bars and results from cells exposed to the SBCMV clinical isolate are shown as stippled black bars. Results are included for **(A)** MIP-1α, **(B)** MMP3, **(C)** MMP9, **(D)** IL-6, and **(E)** IL-8 **(F)** SCF-1, **(G)** GMCSF and **(H)** TNF-alpha. Results are given in pg/ml. Results shown are the averages of triplicate samples.

Over time we have observed that PM from ScienCell can support the growth of human brain vascular pericytes, astrocytes, and brain microvascular endothelial cells, making it an ideal starting point for a universal medium to cultivate the Tricell mixture of human retinal pericytes, retinal capillary endothelial cells (Cell Systems, Kirkland, WA, USA), and primary human Müller cells. We showed that retinal capillary endothelial cells can be cultivated in PM along with retinal pericytes and that the Müller cell line can also be cultivated in PM media (Figure 9A). We found that the Tricell mixed retinal culture had a > 95% viability after 10 days in culture with PM media (Figure 9B). Validation of the Tricell IBRB model was achieved by co-cultivation of the cell mixture, with triple stained IHC for VWF for retinal capillary endothelial cells, NG2 proteoglycan (neuron-glial antigen2) for retinal pericytes, and GFAP glial fibrillary acidic protein for Müller cells (Figure 9C). Supernatants from the Tricell mixed culture exposed to SBCMV, heat-killed virus and media only (mock infected) were also examined by Luminex analysis at 96 hours post infection (Figure 10). We observed a higher level of MIP-1α, MMP9, IL-6 and stem cell factor (SCF-1), no change in MMP3 levels and a lower levels of IL-8, GMCSF and TNF-alpha in SBCMV-infected Tricell cultures compared to mock infected controls (Figure 10). In Tricell cultures exposed to heat-killed virus we observe increased levels of MIP-1α, IL-6, SCF-1, and TNF-alpha, no change in MMP9, and a lower level of IL-8 when compared to mock infected control cultures. In addition, Tricell cultures exposed to heat-killed virus showed higher levels of IL-8, GMCSF and TNF-alpha compared to Tricell cultures exposed to SBCMV.

The Tricell mixture was cultivated in chamber slides, then infected for 96 hours with SBCMV and separately stained pairwise for both MIE or pp28, and the cell-specific markers noted above. Cells co-stained for MIE or pp28 and the respective IBRB cell type-specific markers were counted and the results are shown in Table 2. Both MIE and pp28 co-stained with IBRB cell-specific markers revealed that there was a 10-fold increase in retinal pericyte infectivity for HCMV infection when compared to retinal microvascular endothelial cells and Müller cells (Table 2).

**Table 2:** **Human cytomegalovirus (HCMV)-infected cells in a Tricell retinal model expressing cell type-specific antigenic biomarkers**

**Cells**	**Total MIE(+) cells per 30 fields (200 x)**	**Total cells stained for specific marker**	**Total not stained with specific marker**
Retinal EC	69	5 VWF+	64
Retinal pericytes	86	80 NG2+	6
Müller cells	68	7 GFAP+	61
Cells	Total pp28 (+) cells per 30 fields (200x)	Total stained for specific marker	Total not stained with specific marker
Retinal EC	78	4 VWF+	74
Retinal pericytes	89	84 NG2+	5
Müller cells	93	7 GFAP+	86

*Abbreviations:* *EC* endothelial cells, *GFAP* glial fibrillary acidic protein, *MIE* major immediate protein, *NG2* neuron-glial antigen 2, *VWF* von Willebrand factor.

## Discussion

**Figure 11:**
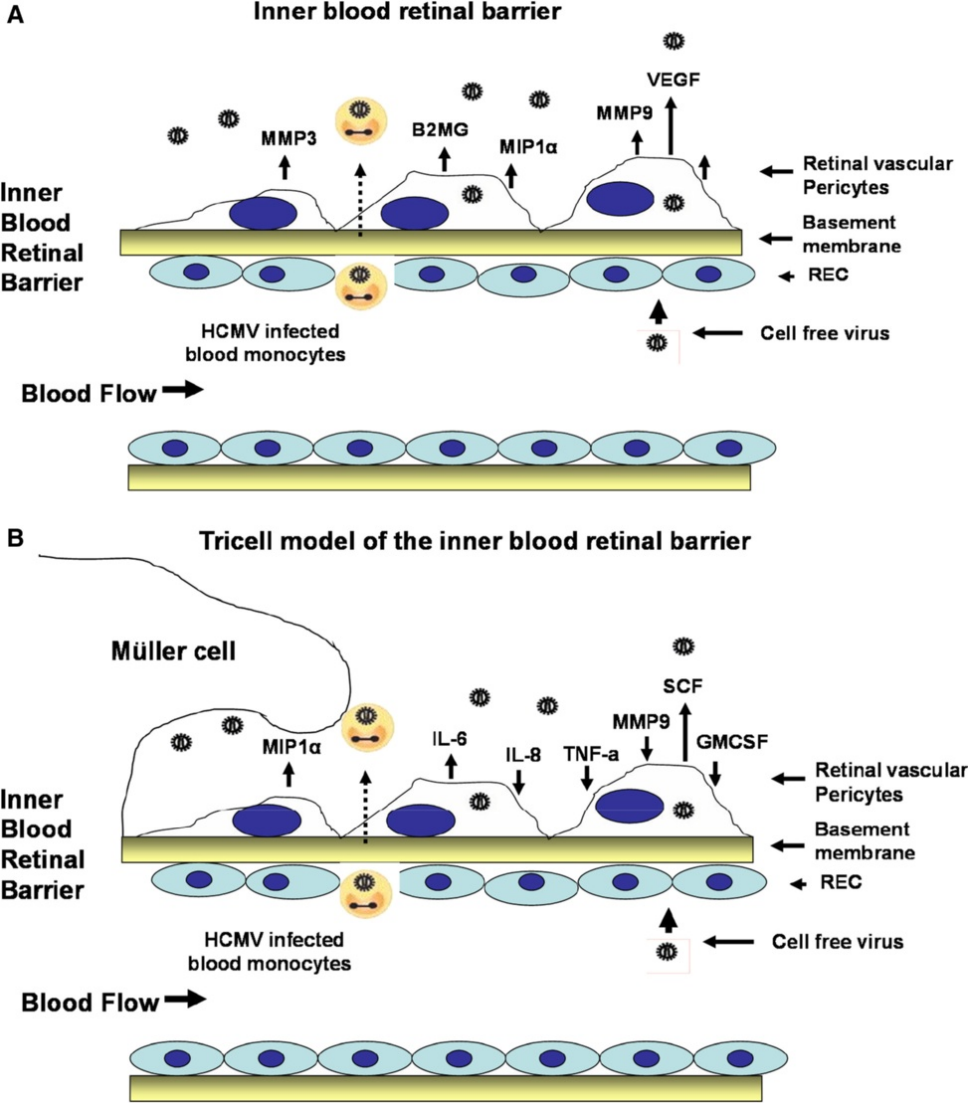
**Working disease models of human cytomegalovirus (HCMV) dissemination across the inner blood-retinal barrier (IBRB).** The models include infected retinal pericytes and a HCMV-induced angiogenic and proinflammatory cascade at late-stage infection **(A)**; Infected Tricell mixture and HCMV-induced dysregulation of angiogenic and proinflammatory cytokines at late-stage infection **(B)**.

The expression profiles of normal human brain and retinal pericytes are shared with respect to several cytoskeletal, cellular adhesion and proinflammatory biomarkers. This suggests that pericytes from different vascular beds within the CNS are similar and that their physiology may be governed by their respective microenvironments. We found that brain and retinal pericytes were equally permissive for HCMV lytic replication by both laboratory adapted and clinical strains of virus. In IBRB, retinal pericytes were most permissive for HCMV infection when compared to retinal microvascular endothelial cells and Müller cells. HCMV infection elicited an angiogenic and proinflammatory cytokine response in pericytes after infection. From these studies we proposed a disease model (Figure 11) for HCMV dissemination across the IBRB into the retina that is similar to the model we proposed for HCMV dissemination across the BBB into the brain [20]. Our working model is that as HCMV traffics the IBRB initially, there is marginal infection of retinal microvascular endothelial cells that poorly supports dissemination of the virus into the barrier. However, HCMV-infected mononuclear cells in the retinal vasculature may gain access through the IBRB via chemotactic signaling by the expression of monocyte chemotactic protein-1 (MCP-1) (Figure 11A). Cell free virus may breach the inner retinal barrier via pinocytosis or paracellular transport. Initial infection of retinal pericytes in a short time period results in a more robust infection (Figure 7) compared to retinal endothelial cells and Müller cells, which results in viral dissemination within the barrier (Figure 9). Pericytes then serve as an amplification reservoir for viral dissemination into ocular tissue. Upon infection, retinal pericytes elicit the angiogenic cytokine VEGF that would likely contribute to retinal angiogenesis and support retinal neovascularization [31-34]. Macrophage inflammatory protein-1 (MIP-1α/CCL3), which was highly expressed in our retinal pericytes after infection, would serve to heighten the inflammatory microenvironment to establish a persistent inflammatory state (Figure 11A). MIP-1α secretion would be chemotactic for monocytes, lymphocytes, and natural killer (NK) cells [35]. MIP-1α has been shown to be induced after HCMV infection and is essential for NK cell migration and IFN-gamma production to mediate antiviral responses in infected cells [36]. High levels of MIP-1α have been observed in gingival fibroblasts infected with HCMV and are thought to play a role in viral pathogenesis linking HCMV infection to periodontal disease [37]. Increased levels of MIP-1α have also been observed in HCMV pulmonary disease in lung transplant patients and have been associated with decreased survival in lung transplant recipients [38]. In addition, an increased level of MIP-1α was observed in blood from HCMV-infected renal transplant patients, a finding that positively correlated with pp65 antigenemia, which was shown to be abrogated by ganciclovir therapy [35]. We observed high levels of beta-2microglobulin (B2m) expression in 9-day HCMV-infected retinal pericytes. In a number of studies, B2-m levels have been shown to have a predictive value for HCMV congenital disease and have been used as a biomarker for fetal distress [39]. Levels of B2-m in cerebrospinal fluid along with neuroimaging have been shown to be of prognostic value for neurodevelopmental outcomes in newborns with HCMV-induced congenital disease [40]. High levels of B2-m in fetal blood were also found to be an important prognostic marker of symptomatic HCMV-induced congenital disease [41]. We also found an increase in the secretion of MMP3 and MMP9 at 9 days post HCMV infection that we propose would aid the virus in tissue dissemination from infected pericytes as it traffics through the inner retinal barrier. HCMV has been shown to induce MMP1 and MMP3 in human aortic smooth muscle cells, which has implications for HCMV-induced plaque inflammation in atherosclerotic disease [42]. Tear MMP9 levels have been shown to be a marker for diagnosing dry eye and ocular surface disease due to inflammation; thus, tear analysis may serve as a gauge to monitor therapy after eye surgery [43,44]. Surprisingly, we observed a lower level of IL-6 and IL-8 in supernatants of 9-day SBCMV-infected pericytes compared to uninfected controls. This is likely due to viral-specific suppression during late-stage infection that would be consistent with chronic HCMV disease. It is has been reported that IL-6 levels are suppressed during active infection in human fibroblasts via transcriptional activation in part by HCMV IE2 protein and posttranscriptional destabilization of IL-6 mRNA [45]. Suppressive effects outweighed transcriptional activation that resulted in less IL-6 production in cells undergoing productive infection compared to controls [45]. In contrast, Luminex analysis of retinal pericytes exposed to SBCMV at the earlier 24 hour time point, revealed increases in IL-8, TIMP-1 and RANTES compared to media only. A significant decrease in MMP9 was observed compared to media controls; however, this is likely due to the increased levels of TIMP-1. We also observed a greater increase in IL-8 in supernatants from retinal pericytes exposed to heat-killed virus at 24 hours compared to SBCMV and media-only-exposed cells. This may be due to virus-specific gene effects on IL-8 expression. Finally, exposure of the Tricell mixed culture to virus for 96 hours, a time of heightened virus replication, revealed a significant increase in the secretion of IL-6 and SCF-1, lower levels of granulocyte-macrophage colony-stimulating factor (GMCSF) and TNF-alpha and a marked increase in MMP9 (Figure 10, 11B). Studies show that a productive HCMV infection reduced MMP9 activity in human macrophages, a finding that was associated with immediate early or early gene expression of HCMV [46]. It has been demonstrated that cmvIL10 inhibited NFkB activation via a reduced degradation of IkappaB-alpha resulting in a decrease in transcription of NFkB responsive genes TNF-alpha and IL-1beta [47]. The decrease in TNF-alpha levels was observed in supernatants from SBCMV-infected pericytes but higher levels of TNF-alpha are observed in pericytes exposed to heat-killed virus that would not express vcmvIL-10 (Figure 10H). Matlaf *et al*., have shown that the HCMV pp71/UL82 protein expressed in human glioblastoma promotes proangiogenic signaling by induction of SCF-1 and that overexpression of pp71 in glial cells also results in an increased expression of SCF-1 [48]. Carleir *et al*., observed both an increase in IL-6 and a concomitant decrease in the expression of GMCSF in dendritic cells derived from HCMV-infected monocytes [49]. They showed that GMCSF signaling was impaired along with a decrease in the phosphorylation of signal transducer and activator of transcription 5 (STAT-5). These cells were unable to stimulate TH1 differentiation and proliferation due to the increased levels of IL-6 that were required for suppressor of cytokine signalling 3 (SOCS3) signaling [49].

## Conclusion

Taken together these studies support the notion that HCMV infection of retinal pericytes is a lytic infection that would result in pericyte loss at proximal sites within the IBRB. This would have implications for retinal neurovascular permeability, and would support an increase in retinal inflammation and angiogenesis. Pericytes appear to be the most permissive cell type within the brain and the inner retinal neurovascular bed that is trafficked by HCMV. Thus, therapeutic modalities designed to specifically protect pericytes from infection could limit HCMV-induced clinical CNS disease. The Tricell culture infection model of the IBRB also has implications for other retinal vasculopathies. An association was previously observed between high HCMV IgG titers and neovascular age-related macular degeneration (AMD), compared to both the dry form of AMD and control subjects [50]. It has been suggested that chronic HCMV infection could be a novel risk factor for the progression from the dry form of AMD to the neovascular or wet form of AMD [50]. Development of novel biological systems that closely mimic the IBRB is essential, as systemic therapies for retinal diseases are highly restricted by the IBRB. Hence it is necessary to develop IBRB model systems to better understand the mechanisms involved in progressive retinopathies and to provide more efficient methods for a timely evaluation of systemic therapies that must circumvent the IBRB [20].

### Consent (Adult)

Written informed consent was obtained from the patient for the publication of this report and any accompanying images and is compliant with standard IRB protocol.

## Abbreviations

SMA: smooth muscle actin
AMD: age-related macular degeneration
B2-m: beta-2 microglobulin
BBB: blood-brain barrier
BMVEC: brain microvascular endothelial cells
CCD: charge-coupled device camera
cDNA: DNA copy of mRNA after reverse transcription
CD68: cluster designation 68 macrophage marker
HCMV: cytomegalovirus
CNS: central nervous system
DAB: 3,3-diaminobenzidine
DAPI: 4′,6-diamidino-2-phenylindole
E-selectin: endothelial-leukocyte adhesion molecule 1
FITC: fluorescein isothiocyanate
GAPDH: gyceraldehyde 3-phosphate dehydrogenase
GFAP: glial fibrillary acidic protein
GFP: green fluorescent protein
GMCSF: granulocyte-macrophage colony-stimulating factor
HCMV: human cytomegalovirus
HFF: human foreskin fibroblast
ICAM-1: intercellular adhesion molecule 1
IFN: interferon
IgG: immunoglobulin-G
IHC: immunohistochemistry
IL: interleukin
mAb810: monoclonal antibody to human cytomegalovirus major immediate early proteins 1 and 2
IBRB: inner blood-retinal barrier
MelCAM-1: melanoma cell adhesion molecule-1
MMP: matrix metalloproteinase
MCP-1: monocyte chemotactic protein-1
MIE 1 and 2: human cytomegalovirus major immediate early gene/proteins 1 and 2
MIP-1α: macrophage inflammatory protein-1
moi: multiplicity of infection
NF: nuclear factor
NG2 proteoglycan: neuron-glial antigen 2
NK cells: natural killer cells
PBS: phosphate-buffered saline
PECAM-1: platelet endothelial cell adhesion molecule-1
PM: Pericyte Medium
pp28: human cytomegalovirus phosphorylated nuclear protein expressed at late times during virus replication
pp65: human cytomegalovirus phosphorylated envelop protein expressed at late times during virus replication
pp71: human cytomegalovirus phosphorylated envelop protein expressed at late times during virus replication
RANTES: regulated upon activation normal T cell expressed and presumably secreted
RT-PCR: reverse transcription polymerase chain reaction
SBCMV: primary HCMV isolate from a patient
SCF-1: stem cell factor
SOCS3: suppressor of cytokine signalling 3
STAT-5: signal transducer and activator of transcription 5
TIMP-1: tissue inhibitor of metalloproteinase-1
TNF-alpha: tumor necrosis factor-alpha
VE-cadherin: vascular endothelial cadherin
VCAM-1: vascular cell adhesion molecule-
VEGF: vascular endothelial cell growth factor
VWF: von Willebrand factor
